# National health insurance policy in Nepal: challenges for implementation

**DOI:** 10.3402/gha.v8.28763

**Published:** 2015-08-21

**Authors:** Shiva Raj Mishra, Pratik Khanal, Deepak Kumar Karki, Per Kallestrup, Ulrika Enemark

**Affiliations:** 1 Nepal Development Society, Chitwan, Nepal; 2 School of Population Health, University of Western Australia, Perth, Australia; 3 Department of Community Medicine and Public Health, Institute of Medicine, Maharajgunj Medical Campus, Kathmandu, Nepal; 4 Nepal Health Economics Association (NHEA), Kathmandu, Nepal; 5 Department of Public Health, Centre for Global Health, Aarhus University, Aarhus, Denmark

**Keywords:** health insurance, poor, premium, access to care, Nepal

## Abstract

The health system in Nepal is characterized by a wide network of health facilities and community workers and volunteers. Nepal's Interim Constitution of 2007 addresses health as a fundamental right, stating that every citizen has the right to basic health services free of cost. But the reality is a far cry. Only 61.8% of the Nepalese households have access to health facilities within 30 min, with significant urban (85.9%) and rural (59%) discrepancy. Addressing barriers to health services needs urgent interventions at the population level. Recently (February 2015), the Government of Nepal formed a Social Health Security Development Committee as a legal framework to start implementing a social health security scheme (SHS) after the National Health Insurance Policy came out in 2013. The program has aimed to increase the access of health services to the poor and the marginalized, and people in hard to reach areas of the country, though challenges remain with financing. Several aspects should be considered in design, learning from earlier community-based health insurance schemes that suffered from low enrollment and retention of members as well as from a pro-rich bias. Mechanisms should be built for monitoring unfair pricing and unaffordable copayments, and an overall benefit package be crafted to include coverage of major health services including non-communicable diseases. Regulations should include such issues as accreditation mechanisms for private providers. Health system strengthening should move along with the roll-out of SHS. Improving the efficiency of hospital, motivating the health workers, and using appropriate technology can improve the quality of health services. Also, as currently a constitution drafting is being finalized, careful planning and deliberation is necessary about what insurance structure may suit the proposed future federal structure in Nepal.

Health policy development in Nepal has been profoundly influenced by the 1978 Alma Ata declaration emphasizing the provision of community-oriented preventive, promotive, and curative health services (1) as evident by the establishment of a network of primary health care facilities and deployment of community health workers to provide essential health services at the community level (2). However, the health system in Nepal faces daunting challenges such as unequal distribution of health care services, poor infrastructures, inadequate supply of essential drugs, poorly regulated private providers, inadequate budget allocation for health, and poor retention of human resources in rural areas. Nepal has only 0.67 doctors and nurses per 1,000 population, which is significantly less than the World Health Organization's recommendation of 2.3 doctors, nurses, and midwives per 1,000 population (3). After restoration of democracy in 1991 and liberalization of the economy thereafter, private health facilities have emerged massively. Within only the last 8 years, nearly two-thirds of the country's total private hospitals have been established (4). The private sector grew from a total share of 23% of all hospitals in 1995 to 78% in 2008. Similarly, private hospital beds are nearly doubled than that of public hospital beds (5) and are unevenly distributed across the regions; that is, the central region – the most developed region – has 76% of the total share, whereas the far western development region – the least developed region – has virtually no private hospitals (5). In terms of total health expenditure, the private sector accounts for 70%, of which 81% comes from out-of-pocket payment (6). Private pharmacies appear to provide the bulk of services covered through private providers. With regards to coverage of essential medicine, the free public health care service initiated by the public sector in 2007 covers only basic health services with 40 essential drugs; for other services, people have to pay out of their pockets and often rely on private health facilities. Out-of-pocket expenditure has remained the principal means of financing health care in Nepal (7).

Nepal's Interim Constitution of 2007 addresses health as a fundamental right, stating that every citizen has the right to basic health services free of cost (8). But the reality is a far cry. Only 61.8% of the Nepalese households have access to health facilities within 30 min, with significant urban (85.9%) and rural (59%) discrepancy (9). The decreasing health budget over the last 5 years shows that Nepal needs to find new ways to increase health care financing (10). Addressing barriers to health services needs urgent interventions at the population level. Recently (February 2015), the Government of Nepal formed a Social Health Security Development Committee as a legal framework to start implementing a social health security scheme (SHS) (technically considered as social health insurance). The insurance scheme aims to ensure universal health coverage by increasing access to, and utilization of, quality health services (11). The first phase of the SHS scheme has been planned to start in three districts (Kailali, Baglung, and Ilam) in 2015, but the details of the SHS design and regulations for implementation are yet to be made public.

Evaluation of Nepal's earlier community-based health insurance (CBHI) schemes showed that CBHI introduced in Nepal since the 1970s suffered from low enrollment and retention of members as well as from a pro-rich bias (12). It is important to learn from this experience. Stoermer (12) provided specific recommendations for Nepal to achieve a more comprehensive national health insurance system: 1) increase the population coverage through a strengthened integrated provincial or national insurance system against the much isolated local insurance system with local capacity of the past; 2) ensure equitable protection for the poor through fair identification mechanisms for enrollment and subsidies; 3) build up efficient ‘voice’ mechanisms through institutional arrangement so that health insurance represents the interests of the insured toward health care providers, and 4) ensure financial viability so that the insurance does not have to rely solely on the premium; government payments may contribute to member's premium payments.

The health insurance policy came as an effort to reduce impoverishment and catastrophic health expenditure, acknowledging that the current system of health care cannot fully identify and protect the poor. However, insurance contributions and copayments can similarly be a barrier for access to insurance, and it is critical to ensure easy enrollment of the poor and marginalized population into the SHS scheme. Various options need to be explored.

With very clear understanding of the health care needs of the Nepalese people and available financial prospects, the overall scheme of SHS including the benefit package can be crafted to include coverage of major health services including non-communicable diseases. Also, engaging the private sector as service ‘providers’ for the health insurance scheme, as envisioned in the policy, needs clear regulations and fair pricing for all services to be covered by the insurance scheme to ensure quality and sustainability as well as to make participation attractive to private providers. Regulations should include such issues as accreditation mechanisms for private providers, specification of minimum benefits to be provided to those insured, pricing control and reimbursement mechanism, protection for poor and vulnerable groups in private care, and monitoring mechanisms.

In essence, the whole idea of insurance is to pool the risks of a large number of people and share the financing of adverse events that strike at random, through prepayment of a contribution, so that no or limited payment is required at point of care when needs arise. This results in a redistribution of resources from those who stay healthy to those who become sick. Low enrollment and retention puts the sustainability of the scheme at risk and reduces the services that can be included in the benefit package. Mandatory contributions to an SHS scheme is therefore preferable, but is a major challenge to implement in countries with a large informal sector. Voluntary enrollment further entails a risk that only those who need the service enroll, which also defeats the purpose of sharing risks. Careful design can to some extent reduce, but not eliminate this risk.

The primary health care system in Nepal has an extensive network with at least one health facility in each village development committee with female community health volunteers in the frontline. However, without focusing on further strengthening of the peripheral health system and ensuring equitable distribution of health services, the government's intention to implement health insurance might not be sufficient for improving access to quality health services that are responsive to people's need. Therefore, health system strengthening should move along with the roll-out of SHS by strengthening demand and supply side. However, Nepal is taking a risky approach by moving toward health insurance without having strong supply and demand side. Countries in transition to insurance programs have made reforms on health financing, such as increasing tax revenues to subsidize target populations, broader risk pools, and emphasis on channeling pooled resources for delivery of care through demand-side and/or supply-side financing mechanisms (13). In addition, the World Health Report 2010 highlights that in removing barriers to accessing health care, elimination of direct payments is necessary but is not sufficient alone; costs of transportation and loss of income can have more impact than direct payment of services (14). This can, for example, be addressed by providing refunds for transportation cost, conditional cash transfer, and microcredit which allow poor households financial assistance to be used for seeking health services.

Demand for health insurance membership cannot be delinked from the quality of health services which the scheme gives access to. Membership will be less attractive if services are of poor quality. Currently, inefficiency of health services is a particular problem, as 20–40% of resources spent in health are wasted which could rather be used in achieving universal coverage. Improving the efficiency of hospitals, motivating the health workers, using appropriate technology, and early and prompt care can significantly improve the quality of health services (14). A review of the free health care program showed the need for improvement of the Nepalese health system in areas such as drug availability, human resources for health, and quality (15). Thus, this suggests that a comprehensive approach should be taken in which the quality of the health care system is improved simultaneously with roll-out of the SHS.

We suggest that more debate and deliberations are needed on how to implement national health insurance policy effectively in Nepal. The prepayment mechanism is still not clear and how much the state will contribute to the pool is still not decided. Looking at the proposed future federal structure of the country, as the constitution drafting process is currently being finalized, a great deal of deliberation is necessary to address what insurance structure will suit the future for Nepal. Furthermore, we urge to plan for a process and outcome evaluation within a year or two of the implementation of the SHS to allow adjustment of the scheme based on identified strengths and weaknesses toward the path of universal health coverage.
